# Supplementary material for: Journey of the tuberculosis patients in India from onset of symptom till one-year post-treatment
Source: PLOS Glob Public Health. 2023 Feb 10;3(2):e0001564. doi: 10.1371/journal.pgph.0001564 (PMC7614204; doi:10.1371/journal.pgph.0001564)
Supplement: S1 Table — (DOCX) [file pgph.0001564.s001.docx]

**S1 Table. States stratified based on levels of development and regions**

|  | Levels of development | | | | | |
| --- | --- | --- | --- | --- | --- | --- |
| Regions | I (Most developed) | II | III | IV | V | VI (least developed) |
| North | Delhi | Himachal Pradesh, Punjab | Haryana, Uttarakhand |  | Jammu & Kashmir |  |
| Central |  |  |  |  | Chhattisgarh | Madhya Pradesh, Uttar Pradesh,  Rajasthan |
| East |  |  | **West Bengal** |  | Odisha,  Jharkhand | Bihar |
| North-east | Sikkim | Mizoram  Tripura | Manipur | Nagaland  Arunachal Pradesh | Meghalaya | **Assam** |
| West | **Maharashtra**  Goa |  |  | Gujarat |  |  |
| South | Kerala | **Tamil Nadu** | Karnataka | Andhra Pradesh |  |  |
