# Supplementary material for: Journey of the tuberculosis patients in India from onset of symptom till one-year post-treatment
Source: PLOS Glob Public Health. 2023 Feb 10;3(2):e0001564. doi: 10.1371/journal.pgph.0001564 (PMC7614204; doi:10.1371/journal.pgph.0001564)
Supplement: S2 Table — (DOCX) [file pgph.0001564.s002.docx]

**S2 Table. Descriptive statistics of explanatory variables in regression analysis**

| Explanatory variables | General population (N = 220) | Tea garden families  (N = 379) | Urban slum dwellers (N = 104) |
| --- | --- | --- | --- |
| Percentage of study participants had outstanding loan in the post-treatment period | 42.53% | 29.02% | 40.38% |
| Average outstanding loan amount – US$ (SD) | 97.42 (214.58) | 29.86 (98.73) | 70.71 (141.31) |
| Average direct treatment cost of tuberculosis – US$ (SD) | 252.86 (259.48) | 97.24 (121.89) | 218.70 (219.91) |
| Average monthly household income in the post-treatment period – US$ (SD) | 200.08 (204.38) | 101.14 (77.12) | 132.18 (81.83) |
| Average age of study participants – years (SD) | 36 (14) | 34 (13) | 33 (14) |
| Percentage of male participants | 69.10% | 57.52% | 51.92% |
| Average cost of treatment in the post-treatment period – US$ (SD) | 23.74 (169.38) | 17.26 (121.93) | 25.02 (202.66) |
| Percentage of study participants borrowed / sold /mortgaged personal belongings in the post-treatment period | 33.03% | 29.55% | 30.48% (N = 105) |
| Average amount borrowed / sold / mortgaged – US$ (SD) | 68.40 (190.04) | 30.24 (88.84) | 47.15 (113.72) (N=105) |

Note: one participant from general population was dropped from the regression because of implausible values of outstanding loan and borrowing/sold/mortgaged and one participant from urban slum dwellers was dropped because of implausible value of outstanding loan.
