# Supplementary material for: Journey of the tuberculosis patients in India from onset of symptom till one-year post-treatment
Source: PLOS Glob Public Health. 2023 Feb 10;3(2):e0001564. doi: 10.1371/journal.pgph.0001564 (PMC7614204; doi:10.1371/journal.pgph.0001564)

**S4 Fig. Fitted line plots with amount of borrowing/selling as dependent variable (Tea garden families, N = 379)**


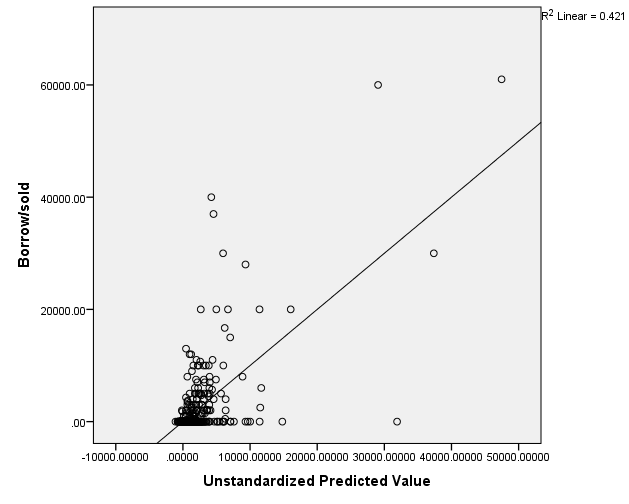

Supplement: S4 Fig — (DOCX) [file pgph.0001564.s006.docx]
