# Supplementary material for: Journey of the tuberculosis patients in India from onset of symptom till one-year post-treatment
Source: PLOS Glob Public Health. 2023 Feb 10;3(2):e0001564. doi: 10.1371/journal.pgph.0001564 (PMC7614204; doi:10.1371/journal.pgph.0001564)

**S5 Fig. Fitted line plots with amount of outstanding loan as dependent variable (Urban slum dwellers, N = 104)**


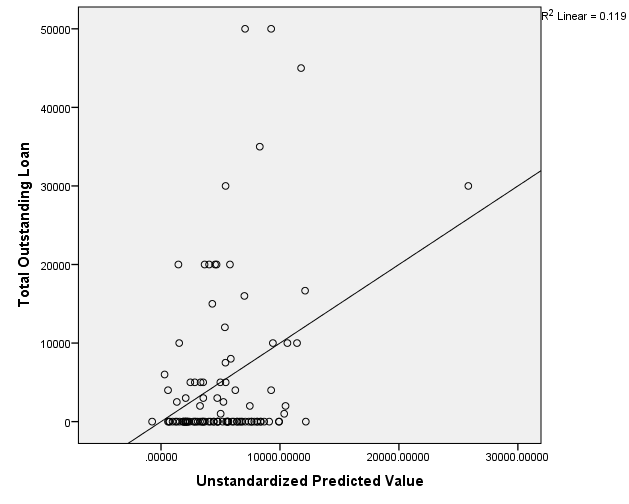

Supplement: S5 Fig — (DOCX) [file pgph.0001564.s007.docx]
