# Supplementary material for: Journey of the tuberculosis patients in India from onset of symptom till one-year post-treatment
Source: PLOS Glob Public Health. 2023 Feb 10;3(2):e0001564. doi: 10.1371/journal.pgph.0001564 (PMC7614204; doi:10.1371/journal.pgph.0001564)
Supplement: Attachment — Submitted filename: Response to Reviewers.docx [file pgph.0001564.s010.docx]

| **Reviewer #1** |  |
| --- | --- |
| **Comments** | **Author responses** |
| A very relevant paper. The economic burden of Non-Communicable Diseases (NCDs) critical issue that needs to be studied more and more. The paper is well structured. However, the authors need to work on the following: | Thank you very much for reviewing the paper and very helpful comments. Below are our responses to each comment |
| The two outcome variables: outstanding loan amount in the post treatment period and the amount of borrowing and sale/mortgage of belongings in the post-treatment period are basically one element of copying mechanisms: Borrowing. Why have you not used other dimensions of copying mechanisms? Why only focus on borrowing indicator while there’s also dissaving (use of savings to finance health care) and sale of property especially, those tied to livelihoods? It is not clear how you combined the amount of borrowing and sale/mortgage of belongings to have one qoutcome variable. | We completely agree with you that use of savings is another coping strategy and we included that as a strategy during treatment period. Participants reported that whatever savings they had were mostly used during the intensive phase of treatment (please see Table 4 in page number 19) and afterwards, borrowing and sale/mortgage of personal belongings were the only way to cope up with the financial distress. Hence, we did not include savings as a coping mechanism in the post-treatment period.  We investigated the types of items sold/mortgaged including those linked with livelihood and now presented with proportions in Pages 18 and 19, line numbers 384-399.  We asked questions about amount borrowed and amount received from sale/mortgage of personal belongings. Those two amounts were added to get the total amount received from of sale/mortgage. We have clarified this in line numbers 245-246. |
| Besides, outstanding loan amount and amount of borrowing…what’s the difference between the two? What proof do you have that the outstanding loan amount was solely to finance health care?...TB treatment? This would really be a strong assumption. You are now running into estimation issues here… | Outstanding loan amount was the amount of loan that the participants took in pre- and during TB period for TB diagnosis/treatment but could not repay at the time of post-treatment interview. On the other hand, borrowing amount was the amount they borrowed again during the post-treatment period. We have now defined outstanding loan in the methods section (please see line numbers 203-204 in page 9)  We agree with you that the outstanding loan amount could be for other reasons also, however, during the interview patients were specifically reminded to report the amount which was only taken for TB treatment and could not be repaid at the time of interview. We also cross checked the reported outstanding loan amounts in the post-treatment period with the amounts borrowed during treatment period for consistency. We have mentioned this in the text, please see line numbers 214-215 in page 9 highlighted section. |
| Also, binary regression models such as probit and logit would have been a better fit here in my opinion than linear regression models. This was to be determined at the point of developing data collection tools though. | We tried different regression models including logit regression, however, linear regression was found to be the best fit. |
| What is the sample size in this study? | This study covered 829 TB patients of whom 435 were from tea garden areas, 260 from general population and 134 from urban slum dwellers. Please see line numbers 178 to 181 in page 8 under the subheading ‘present study participants’. |
| **Reviewer #2** |  |
| Hello, The manuscript entitled ‘Journey of the tuberculosis patients in India from onset of symptom till one-year post-treatment’ presents data on the during treatment and post-treatment economic impacts on the tuberculosis patients in India. The research work is well conducted with scientific rigor. Overall, the manuscript is well written and provides evidence for policy implementation to reduce healthcare costs. A few minor suggestions for your considerations are listed below: | Many thanks for reviewing the manuscript and providing very helpful comments. Please see below our responses to each comment. |
| 1. Line 110: It will be helpful to provide clear definitions for the two high risk groups included in the manuscript for readers understanding. | Following your suggestion, we have now added two paragraphs clearly mentioning the definitions of two high risk groups and the reasons of their inclusion in this study. Please see line numbers 113-127 of the ‘Methods’ section in page 5 |
| 2. Line 119 to 121: The authors provide the sample size calculations in the supplementary; however, it will be helpful to provide the power calculations in the methods section of the main body of the manuscript, rather than supplementary section. | As per you suggestion, sample size calculation for the main study has now been presented in the main text. Please see line numbers 155 to 177 in pages 7 and 8 under the heading ‘Sample size for each group for the main study’ |
| 3. Line 137 to 145: Add a sub header ‘Impact of COVID 19 on the present study’ or present this impact in the supplement. | We have now added a sub-heading ‘*Impact of COVID-19 on the present study’.* Please see line number 206 in page 9. |
| 4. Line 152-153: Provide a source or prevailing wage rate used for the costs calculations. | We have now added the reference for minimum wage rate for study states. Please see line number 224 in page 9 and reference number 26. |
| 5. Line 194: Add table sub headers for the stratification categories for ‘general population’, ‘tea garden families’, and ‘urban families.’ | We have now added the sub headers for the stratification categories for different participants. Please see revised Table 2 in line number 280 of page 12. |
| 6. Page 21, results section: The authors have used a rsquared method to assess the goodness of fit measure; it would be helpful to provide the fitted line plot or prediction intervals in the supplement. Also, did the authors run a sensitivity analysis? If yes, provide information. | We have now added the fitted line plots in the supplement and mentioned about it in the main text. Please see lines 252-254 in page 11 and the graphs (Figure S1 to Figure S6) in the supplement.  We did not conduct any sensitivity analysis in this paper. |
| 7. Page 27, figure 1A: It is hard for the reader to clearly read the label in the graph. I hope the graphs in the final article are higher in resolution. | We are sorry for the inconvenience. We have now saved all graphs in higher resolution. We hope those look better now. |
| **Reviewer #3** |  |
| An important topic of out-of-pocket expenditure on treatment of TB over one-year period in three types of households are covered. There are several methodological problems in the survey because the conceptual framework on cost of TB treatment has not been described meticulously. | Thank you so much for reviewing the paper and very helpful comments. We have addressed all the comments. Please see below our responses to each comment |
| Both pre-treatment and post-treatment phase the recall period is not defined which affect the types of cost and the ir magnitude, e.g. longer the recall period in the post-treatment phase would lead to higher accumulation of costs. They need to do a rigorous exercise by calculating the number of days the cost details for each household in four phases; pre-treatment, IP, CP and Post-treatments and then relate to the variation in costs. | As per your suggestion, we have calculated the recall period for each phase and linked that with the cost of that period. However, we did not find any correlation between recall period and cost. For example, pre-treatment recall period ranged from 52 days (SD 57) to 54 days (SD 55) and the correlation coefficient ranged from 0.08 to 0.16. Similarly, for the post-treatment period, average recall period ranged from 272 days (SD 75) to 314 days (SD 91) and the coefficient ranged from 0.01 to 0.23.  The reason for this is even though there was a long delay from symptom to treatment initiation, and hence, the long recall period, the patients often did not do anything for weeks. They bought over the counter medicines or made an outpatient visit, got treatment for cough, fever, and felt better and did not seek any other treatment for some time. Hence, the delay did not have impact on cost, rather, the treatment seeking behaviour impacted the cost. However, even if there were recall bias, those were minimized by reviewing medical records. This is mentioned in the limitation section. Please see line numbers 511-513 in page number 27. Even though we did not find any correlation between recall period and cost, we have now mentioned the recall period for each phase in the main text. Please see line numbers 262-266 in page number 11.  Similar in post-treatment period, as mentioned in line numbers 327, 330-331, less than 20% of those suffered from post-treatment sequalae sought treatment and only 4% had recurrent cases – therefore, treatment cost was very low in the post-treatment period ranged from 6%-7% of total treatment cost (please see line numbers 341-342). Hence, recall period did not have impact on the cost. |
| No specific reason is provide to distribute equally the sample between three groups and in the paper they have provided a very highly skewed distribution of cases between these three groups. | We apologise for the incorrect sentence. The sample size was calculated to ensure representative sample of drug-susceptible TB patients for India and the calculation showed we need 512 patients. As we considered three groups for our study, we required 512 in each group to be representative of those groups – hence, we aimed to interview total 1536 patients (512 in each group). We have now corrected this and provided the sample size calculation in the main manuscript instead of appendix. Please see page number 7, line numbers 155 to 177. |
| Some of the variables (also dependent variables) included in the analysis are not very well defined e.g. wealth index, household income reference period, amount of borrowing (whether outstanding at the time of re-visit during post-treatment phase, and its recall period). | We have now defined all these in the text. Household income before TB implied household income before the onset of TB symptom; outstanding loan was defined as the loan the study participants took for TB diagnosis / treatment during pre- and treatment period but could not repay at the time of post-treatment interview. We have also explained in the text how we constructed the wealth index. Please see line numbers 203-204 and 214-215 in page 9 and 239-244 in page 10.  Recall periods for all phases are now added in the text. |
| Recall period for hospitalisation cases has not been conceptualised and defined. | In each phase of interview, hospitalization related questions were asked, and so the recall period was the same as the recall of each phase of interview. |
| At several places statistics are presented without any relative comparison (If one reads some statistics on cost or DALYs one needs to compare with similar disease to see the difference otherwise these statistics are meaningless). | To the best of our knowledge, this is the first study that reported the post-treatment experience of the TB patients in India. Hence, there is no comparative data for Indian context. However, we compared our results with a similar study conducted in Malawi.  We compared our cost estimates and distribution of costs with previously conducted studies. Please see line numbers 456-458 and 472-473 in page 25, 479-480 in page 26. |
| There are several publications on cost of TB in India using National Sample Survey dataset and authors have not touched-upon this neither in the literature search nor in discussion sections. | We agree with you that we did not touch upon the publications on cost of TB treatment from National Sample Survey data. This is because as we collected data from TB patients directly following WHO recommendations on TB patient cost surveys and we compared our results only with those studies where data were collected from the TB patients and followed WHO guidelines. However, for comparing the catastrophic cost because of TB, we cited one recently published paper based on national survey data. Please see line numbers 494-497 in page 26 and reference number 31. |
| Some of the Table titles need proper headings. One can't understand Table 2 because each group have two columns without any headings. | We have now corrected this – please see revised Table 2 in line number 280 in page 12. |
| Most cross-tabulations required statistical testing for differences in three groups. | We have now provided comparison results among three groups in the text. Please see line numbers 277-279 in page 12, 358-359 in page 16. |
| Results interpretation and discussion need re-writing and should be very analytical with policy implications. | We have modified the results and discussion sections as per suggestions from all reviewers. |
| **Reviewer #4** |  |
| This is a very interesting study that estimates patient costs for TB patients in India. While the analysis is neat, it could be extended to increase impact. Some comments for the authors' consideration. | Many thanks for reviewing the manuscript and providing very helpful comments. We have addressed all comments. Please see below our responses |
| Abstract, lines 44-45- It would be good to name the Indian states in which the research was conducted. | As mentioned in the paper, this is an interim analysis with two states data out of four. We did not mention the names of these two states in this paper as we have an agreement with the state officials which says that we will mention names of all four states but will not specifically identify any state. As these data are from two states only and readers may be able to identify the states from the categories of patients, we did not mention the state names here. However, in the sampling strategy, we have mentioned names of all four states and reasons of selection. Please see page number line numbers 129-142 in page number 6. |
| Introduction - this section clearly explains that TB patients bear high costs, and that these are catastrophic but doesn't state why it is important to understand the full extent of the costs, and how this evidence could be relevant for health financing policy solutions or public health/ health systems policy. This weakens the motivation of the paper to some extent. | We have now added a line on this. Please see line numbers 105-107 in page 5. |
| Methods - The section on study design requires further details in the main text, especially which states was this research conducted in, along with some background information to set the frame of reference for the reader. | We have now added those details in the main text. Please see the sampling strategy section in line numbers 128-148 in page number 6 |
| - Lines 127-132 summarise the data collected, which appears to be medical costs only. This should be clearly stated in this section. | We apologize for the confusion. We have now clarified that data were collected on out-of-pocket expenses and time cost for patient and accompanied persons for outpatient visits, hospitalization, drug pick-up etc. As indirect cost was calculated multiplying time spent related to TB with minimum wage rate of respective states, time cost is the indicator of indirect cost here. We have added time cost in both CP and post-treatment interview part as well. Please see line numbers 187-205 in pages 8 & 9. |
| - Non medical costs were not included, understandably because of the complexities introduced by the pandemic. This should be mentioned in the limitations. | We sincerely apologise for not making these clear in the manuscript. We indeed added non-medical costs. We have now added specific cost components added in the calculation. Please see line numbers 187-198 in page 8. |
| - Were these costs regressive? I would imagine that they would be. It would be good to add this analysis to extend the impact of the results. | We have now presented the burden of cost (catastrophic cost) by wealth quintile. Please see line numbers 362-368 and Figure 2 for the same. |
| - Was it not possible to calculate catastrophic expenditure using the Feb 2020 estimates of incomes, with a ceterus paribus assumption, that could have indicated the extent of the impact that costs could have on households? | Based on your suggestion, we have now presented the catastrophic cost, however, we used the definition recommended by WHO, i.e., total treatment cost of TB ≥20% pre-TB annual household income. Please see line numbers 224-226 and line numbers 362-368 for the same. |
| Discussion: The regressively of financing/ coping mechanisms would make this a more compelling section of the manuscript (page 18. Sorry, I can't refer to the line numbers as these disappear after page 13). | We have now added a section on coping strategies by wealth quintile in the results section. Please see line numbers 403-408 and Table 5 in line number 409. In the discussion section also, we spoke about the impact of catastrophe and coping strategies by wealth quintile. Please see line numbers 497-500 and 530-535. |
